# The effects of elevated temperature and dissolved ρCO_2_ on a marine foundation species

**DOI:** 10.1002/ece3.2969

**Published:** 2017-04-18

**Authors:** Cori J. Speights, Brian R. Silliman, Michael W. McCoy

**Affiliations:** ^1^ Department of BiologyEast Carolina UniversityGreenvilleNCUSA; ^2^ Division of Marine Science and ConservationNicholas School of the EnvironmentDuke UniversityBeaufortNCUSA

**Keywords:** acidification, climate change, multiple stressors, oyster, warming

## Abstract

Understanding how climate change and other environmental stressors will affect species is a fundamental concern of modern ecology. Indeed, numerous studies have documented how climate stressors affect species distributions and population persistence. However, relatively few studies have investigated how multiple climate stressors might affect species. In this study, we investigate the impacts of how two climate change factors affect an important foundation species. Specifically, we tested how ocean acidification from dissolution of CO_2_ and increased sea surface temperatures affect multiple characteristics of juvenile eastern oysters (*Crassostrea virginica*). We found strong impacts of each stressor, but no interaction between the two. Simulated warming to mimic heat stressed summers reduced oyster growth, survival, and filtration rates. Additionally, we found that CO_2_‐induced acidification reduced strength of oyster shells, which could potentially facilitate crab predation. As past studies have detected few impacts of these stressors on adult oysters, these results indicate that early life stages of calcareous marine organisms may be more susceptible to effects of ocean acidification and global warming. Overall, these data show that predicted changes in temperature and CO_2_ can differentially influence direct effects on individual species, which could have important implications for the nature of their trophic interactions.

## 1 Introduction

Anthropogenic climate change is dramatically impacting natural ecosystems (Hoegh‐Guldberg & Bruno, 2010; IPCC, 2014, Walther et al., 2002). Increasing greenhouse gas (e.g., CO_2_) (Raynaud et al., 1993) concentrations in the atmosphere and rising surface temperatures are leading to changes in weather patterns and the loss of ice sheets which are contributing to sea level rise and salinity increases in coastal habitats (Nicholls & Cazenave, 2010). Global mean surface temperature is expected to increase by 0.3–4.8°C (IPCC, 2014) by the end of the twenty‐first century, while dissolution of elevated atmospheric CO_2_ into oceans is expected to simultaneously decrease ocean pH by approximately −0.0014 to −0.0024 per year over this same time period (Rhein et al., 2013). Such dramatic changes are expected to significantly impact biodiversity and the normal functioning of ecosystems (Doney et al., 2012), but we still do not fully appreciate which species will be impacted and to what extent these impacts will be manifested (Moritz & Agudo, 2013). For example, changes in species phenology, community composition, and range shifts caused by climate change are altering species distributions and the interaction networks experienced by many species (Walther et al., 2002). While numerous studies have examined how the effects of climate drivers such as temperature, salinity, and pH affect the autecology of individual species (Crain, Kroeker, & Halpern, 2008; Parmesan, 2006), relatively fewer studies have attempted to elucidate how multiple global climate change factors affect both physical and biological interactions (Prugh et al., 2009; Rosenblatt & Schmitz, 2014).

A recent meta‐analysis of 328 studies manipulating at least one climate change variable revealed that multiple stressors often combine to cause larger effects than expected relative to single stressor manipulations (Rosenblatt & Schmitz, 2014). Alternatively, simultaneously changes to multiple global change factors could create counteractive effects. For example, a decrease in pH can impact the growth and strength of individuals with calcium carbonate shells (Ivanina et al., 2013), but with an accompanying increase in temperature, the solubility of carbonate ions is reduced, therefore the negative effects of lowered pH may be ameliorated. Other anthropogenic activities such as overharvesting of higher trophic level predators (e.g., *Callinectes sapidus* and *Menippe mercenaria*) can increase the abundance of mesopredators and indirectly alter trophic and nontrophic interactions (Silliman & Bertness, 2002). Combined, shifts in pH, temperature, and predator abundances are likely to impact the structure and diversity of coastal communities, especially those formed around foundation species (e.g., oysters). We must therefore enhance understanding of how abiotic global climate change variables interact to affect foundation species if we are to better predict and mitigate the longer term consequences of global change on the proper functioning of marine and coastal ecosystems, which are currently understudied in this context (Griffen, Belgrad, Cannizzo, Knotts, & Hancock, 2016; Prugh et al., 2009; Rosenblatt & Schmitz, 2014).

Oysters are autogenic foundation species (Dayton, 1973; Ellison et al., 2005; Jones, Lawton, & Shachak, 1994) that create a structurally complex reef that facilitates other species by providing resources, refugia, and settlement space for sessile individuals (Gutiérrez, Jones, Strayer, & Iribarne, 2003). By serving as a barrier between the coast and the shoreline in many systems, oyster reefs reduce coastal erosion (Meyer, Townsend, & Thayer, 1997), provide water filtration, help reduce eutrophication (Newell, 2004), and function as important nutrient (Smyth, Geraldi, & Piehler, 2013) and carbon sinks (Granek, Compton, & Phillips, 2009; Volety, Haynes, Goodman, & Gorman, 2014; Wingard & Lorenz, 2014). Oysters are also a valuable fishery and serve as nursery habitat for other important fisheries and nonfishery species. In North Carolina, oyster harvest is estimated to generate between $12.80 and $32.00 per 10 m^2^ (Grabowski & Peterson, 2007) Unfortunately, a recent synthesis suggest that in almost 40% of estuaries and bays (of 144 evaluated globally) 99% of the oyster reefs are functionally extinct and thus are not providing ecosystem functions and services (Beck et al., 2011). In North Carolina, for example, tens of millions of dollars is invested in efforts to recover eastern oyster fisheries (Beck et al., 2011) and their biogenically created habitat. However, the long‐term sustainability of such efforts may not be realized if scientists do not understand how multiple climate change stressors impact the health and ecology of oysters specifically.

Investigations into the effects of acidification or sea surface temperatures on eastern oysters show variable results. Elevated temperatures reduce energy reserves and increase mortality of adult oysters, and the combined effects of reduced pH (via increased dissolution of CO_2_) and temperature causes reductions in shell hardness (Ivanina et al., 2013; Matoo, Ivanina, Ullstad, Beniash, & Sokolova, 2013). In contrast, increased temperature has been shown to have no detectable effects on juvenile eastern oysters (Talmage & Gobler, 2011). Elevated concentrations of CO_2_ can negatively impact oyster calcification response (Ries, Cohen, & Mccorkle, 2009), with the larval stage being more vulnerable than the juvenile stage (Talmage & Gobler, 2011). One study on the larval stage of oysters found mineral saturation state conditions to have the largest impact on larval oyster shell formation (Waldbusser et al., 2015). However, juvenile eastern oysters have increased mortality rates in addition to reduced shell growth in low pH environments (Beniash, Ivanina, Lieb, Kurochkin, & Sokolova, 2010).

In this study, we build upon these earlier studies by investigating if elevated CO_2_ and increased temperature will impact juvenile eastern oyster (*Crassostrea virginica*): (1) growth, (2) survival, and (3) filtration rate.

## 2 Methods

### 2.1 Experimental setup

**Figure 1:**
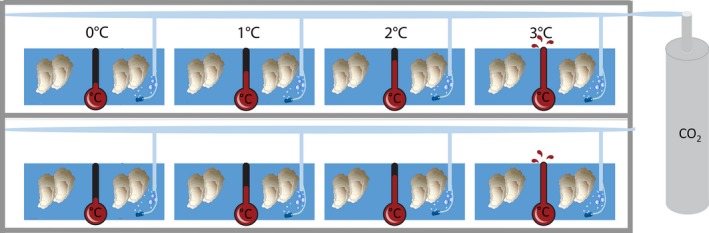
Experimental setup. Oysters (*Crassostrea virginica*) were placed in one of eight possible treatments. There were four temperature treatments (0, 1, 2, 3°C above ambient temperature) that heated two containers each, and one of the two containers received an input of CO_2_ (Note: Each CO_2_ treatment had its own CO_2_ tank) The treatments were each replicated once (top row and bottom row). A total of 60 oysters were placed into each container, totaling 120 oysters for each CO_2_/temperature treatment

This experiment was conducted in a flow‐through aquaculture system at the Duke Marine Laboratory in Beaufort, North Carolina (Figure 1). Unfiltered seawater from Back Sound flowed into 18.9 L buckets arranged in the center of eight 1.22 × 0.61 m bins. Each bucket was equipped with two to three submersible aquarium heaters to maintain desired temperature treatments. Heated water flowed from the buckets into two 5.68 L plastic containers (34.3 × 21.0 × 12.1 cm) containing juvenile oysters (spat). To simulate ocean acidification, CO_2_ was diffused into one of the two paired 5.68 L containers. To maintain pH at the desired level, each tank was outfitted with a dual regulator equipped with a solenoid valve (purchased from Green Leaf Aquariums). The solenoid valve (which allowed gaseous CO_2_ to flow or not flow) was regulated in real time by a pH probe attached to a digital pH monitor. The probe detected the pH of the water and opened or closed the solenoid valve to maintain the pH at 7.8 (the 2081–2100 year RCP8.5 prediction for ocean acidification) (IPCC, 2014). This setup allowed simultaneous manipulation of both temperature and pH (via ρCO2) of continuously flowing unfiltered seawater. Surface water temperature from which flow‐through water was sourced naturally varied, therefore our temperature treatments maintained water temperatures at approximately 0, 1, 2, and 3°C above ambient (Table 1). Ambient temperatures varied from 18.5 to 30.0°C over the duration of the experiment (Table 2). pH probes were calibrated monthly (or on an as needed basis). Temperature and pH were measured twice a day using secondary handheld probes to insure the system was functioning properly.

**Table 1:** Mean ± *SE* for temperature and pH of each treatment tank

Tank ID	Temperature (°C above ambient)	pH
T1A	1.84 ± 0.04	7.80 ± 0.01
T1B	1.86 ± 0.04	8.02 ± 0.01
T2A	0.10 ± 0.02	8.02 ± 0.01
T2B	0.10 ± 0.02	7.83 ± 0.01
T3A	2.88 ± 0.04	8.01 ± 0.01
T3B	2.89 ± 0.04	7.77 ± 0.01
T4A	1.17 ± 0.03	8.01 ± 0.01
T4B	1.13 ± 0.03	7.78 ± 0.01
T5A	1.05 ± 0.04	7.78 ± 0.01
T5B	1.09 ± 0.04	8.01 ± 0.01
T6A	0.24 ± 0.02	7.78 ± 0.01
T6B	0.13 ± 0.02	8.02 ± 0.01
T7A	2.90 ± 0.04	8.01 ± 0.01
T7B	2.99 ± 0.04	7.84 ± 0.01
T8A	1.97 ± 0.03	7.80 ± 0.01
T8B	2.05 ± 0.03	8.01 ± 0.01

Temperatures are displayed as degrees above ambient temperature. Each set of heaters managed two tanks (e.g., T1A and T1B), and within those two tanks one received additional CO_2_ (e.g., T1A) lowering the pH.

**Table 2:** Mean ± *SE* of the ambient temperature and pH for each month during the experiment (2 June 2015–19 October 2015)

Month	Ambient temperature (°C)	Ambient pH
June	28.1 ± 0.22	8.05 ± 0.01
July	28.1 ± 0.09	8.03 ± 0.01
August	27.8 ± 0.1	8.01 ± 0.01
September	26.4 ± 0.19	8.07 ± 0.02
October	23.1 ± 0.26	7.97 ± 0.02

June and October were not complete months.

In May 2015, 1,000 individual oyster (*Crassostrea virginica*) spats were obtained from Millpoint Aquaculture in Sea Level, NC. Individual oysters were pooled into groups of 10 and placed into 24 in. (61 cm) mesh mariculture bags. For each group of ten, we quantified initial wet weight (g) using an electronic balance (Ohaus Valor 3000) with a readability of 0.01 g and photographed (Cannon T5, 55 mm lens) each group to measure oyster height (distance from umbo to dorsal edge) using Image J software (1.44 ± 0.02 cm average). Oyster bags were then randomly assigned to a specific CO_2_/temperature treatment. Six bags of oysters were placed into each experimental arena (total of 60 oysters per container, 120 per treatment) and weighed on a weekly basis. After 2 months, oyster tanks were supplemented with 21.5 ml of a 1/10 dilution of Shellfish Diet 1800 (Reed Mariculture, Inc.). To add the supplemental diet, water flow was briefly stopped (1 hr each day), and oxygen was bubbled into the tanks to keep acceptable DO (dissolved oxygen) levels. After 5 months, all oysters were photographed for height (2.01 ± 0.03 cm average) and survival was quantified.

### 2.2 Oyster shell strength experiment

After 5 months, all remaining oysters were sacrificed and stored at 20°C. Ten individuals from each treatment were randomly chosen for a shell strength assay. Before each assay, we measured height (distance from umbo to dorsal edge) (mm), length (distance between anterior and posterior margin) (mm), and whole oyster shell thickness (largest distance between the outsides of the closed vales) (mm) with digital calipers. To determine the relative force needed to crush each oyster, they were individually placed under a flat metal surface beneath the outer edge of an 18.9 L bucket. Sand was added to the bucket at a slow but continuous rate until the oyster shell was crushed (Osenberg & Mittelbach, 1989). The mass needed to crush the shell (kg) was recorded as a relative measure of shell crushing resistance (an important deterrent of mud crab predation).

### 2.3 Filtration experiment

Approximately 3 months into the experiment, five oysters from each CO_2_/temperature were randomly selected, and wet weights (g) were recorded. Each group of five oysters was placed into a 50 ml nalgene tube for the assay. A tube with no oysters was used as a control for this experiment. The tube was filled with 25 ml of water from each individual tank and 3 ml of Shellfish Diet 1800 (1/10 dilution). The lids were left off to allow oxygen and then left undisturbed for 1.5 hr. After 1.5 hr, the tubes were lightly shaken to insure re‐oxygenation of the water and resuspension of shellfish diet; after 6 hr, 10 ml of tank water was added to the respective tube, and after 7.5 hr the tubes were shaken a second time. To determine the amount of feces produced by oysters, a proxy for oyster filtration, the samples from each tube were run through vacuum filtration using 47 mm glass microfiber filters. After each sample was filtered, all equipment was rinsed in water followed by a 70% ethanol solution. Each sample was run through filtration for five minutes and afterward placed in a 60°C oven for 1 week. Each filter was weighed on an electronic balance before filtration and after drying. This experiment was replicated three times in each of two time blocks separated by ~1.5 months.

### 2.4 Statistics

All data were analyzed in the R statistical programing environment (R Core Team, 2016). For all analyses, we used median temperature from daily measurements as a continuous covariate, and CO_2_ was treated as a two level factor: elevated or ambient CO_2_. To analyze oyster height (mm) and wet weight (g), we used linear mixed effects models (LMM), where CO_2_ and temperature were treated as fixed effects, and tank ID was treated as a random effect to account for autocorrelated errors among individuals reared in the same tank. To analyze oyster survival, we used a generalized linear mixed effects model (GLMM) with a binomial family error distribution. CO_2_ and temperature were treated as fixed effects, and tank ID as a random effect. We also added an individual level random effect to account for mild overdispersion in the data (Bates, Mächler, Bolker, & Walker, 2015). Relative crush force data were analyzed using a linear model (LM). Oyster shell thickness was treated as a continuous covariate in the model. Additionally, oysters were pooled by treatment and then randomly selected for experimentation to insure that any error due to individuals being reared in a common environment was randomly redistributed into the overall residual error for model fits. Filtration data were analyzed using a LMM, where CO_2_ and temperature were treated as fixed effects, and tank ID and time block (one or two) were treated as random effects. Inferences from LMs, GLMs, LMMs, and GLMMs are based on likelihood ratio tests comparing models with and without target fixed effects. Model assumptions were evaluated visually using QQ plots, residual plots, and likelihood profiles, as appropriate.

## 3 Results

### 3.1 Effects of CO_2_ and temperature on oysters

**Figure 2:**
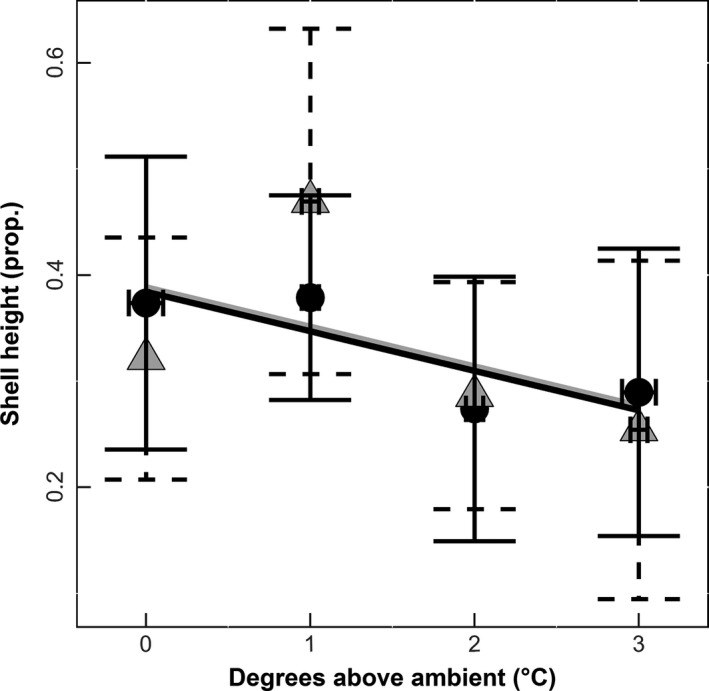
Shell height. Temperature is displayed as degrees Celsius above ambient, and growth is a measure of the log difference between final and initial oyster height (*n* = 92). Lines represent predicted values of either elevated (black) or ambient (gray) CO_2_. Individual points represent the average growth using the raw data elevated (Ο) or ambient (Δ) CO_2_ with horizontal and vertical error bars representing the standard deviations

**Figure 3:**
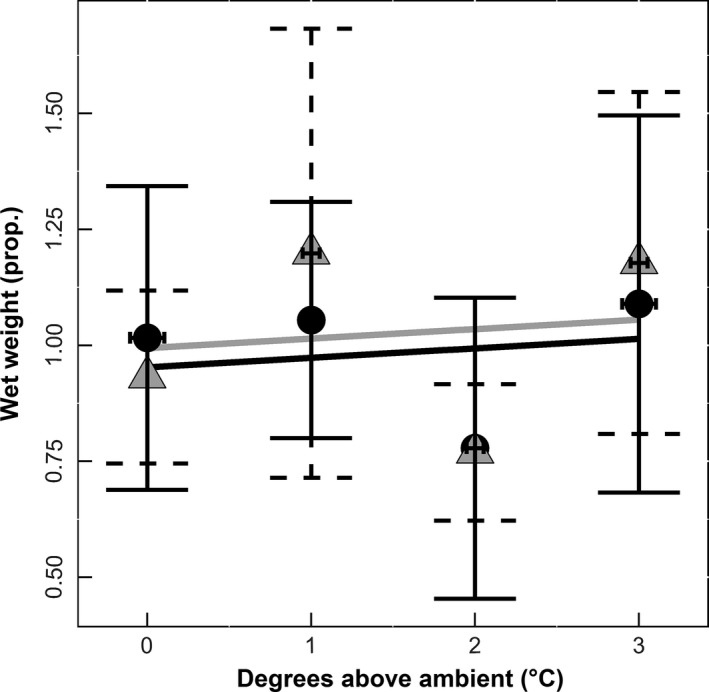
Wet weight. Temperature is displayed as degrees Celsius above ambient, and wet weight is a measure of the log difference between final and initial oyster wet weight (*n* = 92). Lines represent predicted values of either elevated (black) or ambient (gray) CO_2_. Individual points represent the average growth using the raw data elevated (Ο) or ambient (Δ) CO_2_ with horizontal and vertical error bars representing the standard deviations

**Figure 4:**
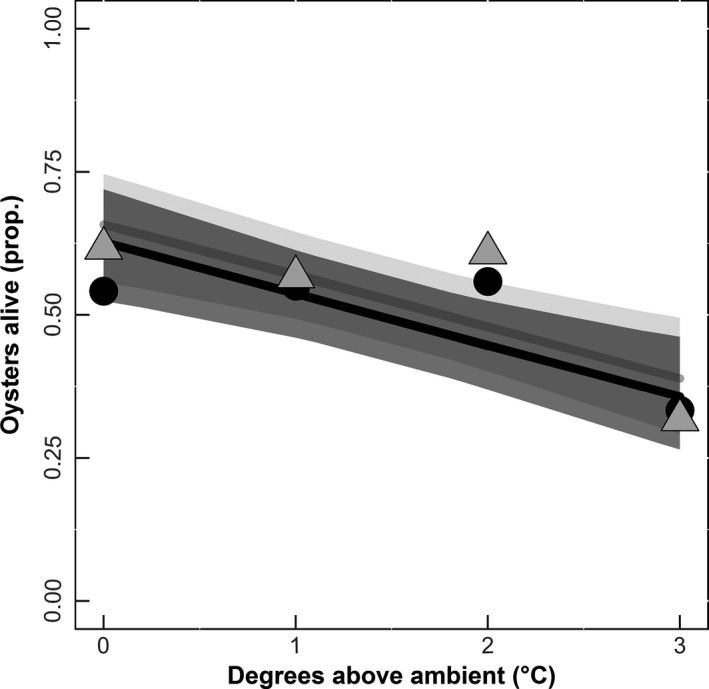
Proportion of oysters alive (before the mud crab (*Panopeus* spp.) predator trials). Temperature is displayed as degrees Celsius above ambient, and survival is the final divided by initial number of oysters alive (*n* = 96). Lines represent predicted values (binomial error distribution) of either elevated (black) or ambient (gray) CO_2_, and the corresponding envelopes represent 95% confidence intervals. Individual points represent the average proportion alive using the raw data for elevated (Ο) or ambient (Δ) of CO_2_

Oyster height (mm) decreased with increasing temperature over the course of the experiment (*df* = 1, χ^2^ = 4.4798, *p* = .034; Figure 2). There was no relationship between oyster wet weight and temperature (*df* = 1, χ^2^ = 0.1586, *p* = .69; Figure 3). However, there was a significant reduction in oyster survivorship (*df* = 1, χ^2^ = 9.584, *p* = .001; Figure 4) with increasing temperature. There was no impact of elevated CO_2_ on oyster height (*df* = 1, χ^2^ = 0.0199, *p* = .88; Figure 2), oyster survival (*df* = 1, χ^2^ = 0.4041, *p* = .52; Figure 4), or wet weight of oysters (*df* = 1, χ^2^ = 0.1717, *p* = .67; Figure 3).

**Figure 5:**
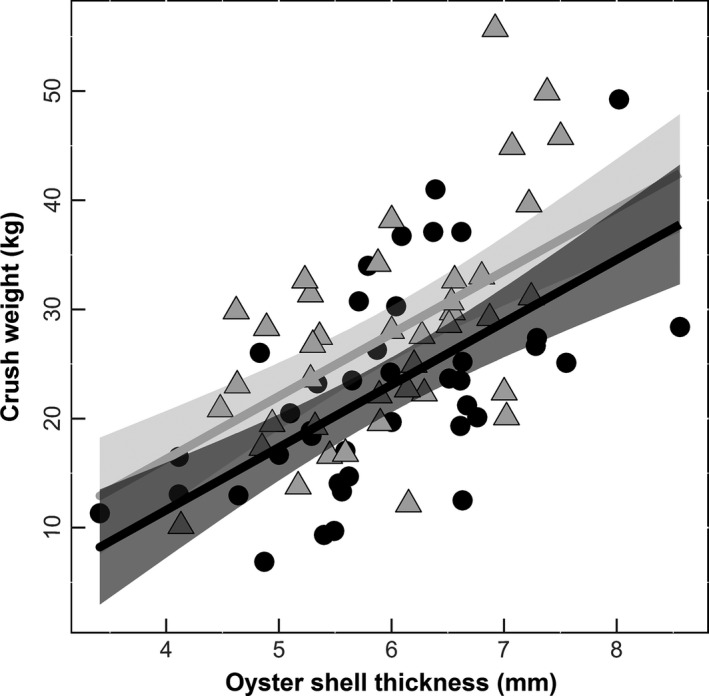
Crush weight (proxy for shell strength). Sand plus bucket weight is represented as crush weight (kg) (*n* = 80). Lines represent predicted values of either elevated (black) or ambient (gray) CO_2_, and the corresponding envelopes represent 95% confidence intervals. Individual points represent the raw data for elevated (Ο) or ambient (Δ) CO_2_

Oysters grown in elevated CO_2_ environments also required significantly less crushing force than oysters in ambient CO_2_ conditions (*df* = 1, F = 6.96, *p* = .01; Figure 5). While whole oyster shell thickness affected the amount of weight to crush oysters (*df* = 1, F = 38.688, *p* < .001; Figure 5), there was no relationship between temperature and crushing force, or temperature and oyster shell thickness.

**Figure 6:**
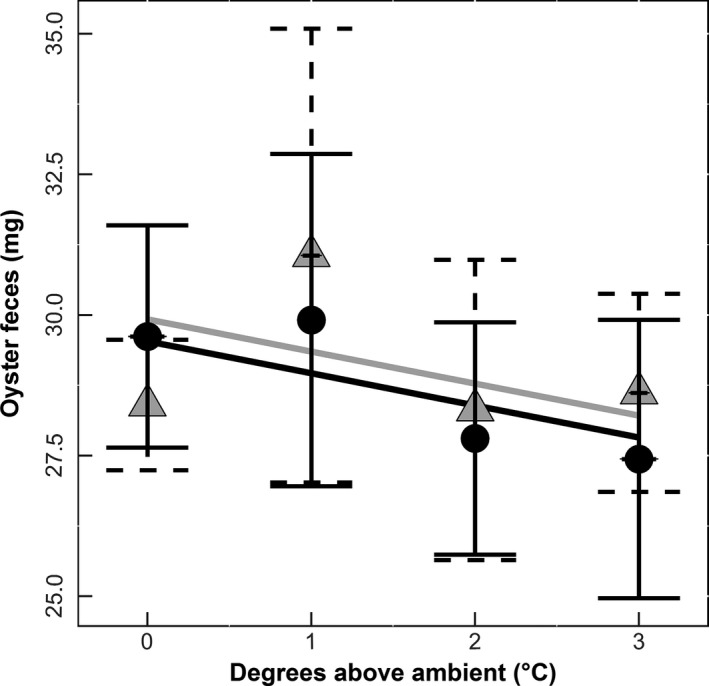
Oyster feces (proxy for filtration). Temperature is displayed as degrees Celsius above ambient, and oyster feces is a measure of the log difference between final and initial filter weight (*n* = 95). Lines represent predicted values of either elevated (black) or ambient (gray) CO_2_. Individual points represent the average growth using the raw data elevated (Ο) or ambient (Δ) CO_2_ with horizontal and vertical error bars representing the standard deviations

Oysters filtered less from the water (as measured by fecal production) as temperature increased (*df* = 1, χ^2^ = 3.9089, *p* = .048; Figure 6). There was no impact of elevated CO_2_ on oyster filtration (*df* = 1, χ^2^ = 0.4902, *p* = .48, Figure 6).

## 4 Discussion

In this study, we found that oysters grown in higher temperatures had decreased growth (Figure 2) and survival (Figure 4). Moreover, oysters grown in elevated CO_2_ environments had weaker shells (Figure 5). By quantifying the effects of temperature and CO_2_ on oysters, we uncovered differential impacts of multiple stressors on these organisms that would have been undetected when focused solely on a single interaction.

The significant decrease in oyster height without changes in shell thickness suggests temperature dependent deposition of carbonate ion placement during shell formation. Indeed, other studies have documented no differences in shell height yet increases in shell thickness as a function of temperature (Lord & Whitlatch, 2014). We also found a significant decline in juvenile oyster survival with increasing temperature. This result contrasts with studies such as Talmage and Gobler (2011) that found no significant decline in juvenile oyster survivorship with an increase in temperature of 4°C. However, this difference may be driven in part by differences in experimental duration (a shorter duration experiment experiences less mortality) or differences in resource availability. In this study, there were low levels of natural food resources in the flow‐through water during the first 2 months of our study that may have affected later sensitivity to increased temperature. Indeed, similar effects and reductions in survival with increases in temperature have been documented in other studies (e.g., Ivanina et al., 2013). Finally, increasing temperature also reduced fecal production by oysters. While decreasing temperatures has been shown to reduce filtration rates of oysters (Walne, 1972), we manipulated temperatures above ambient during the warmest summer months, which may have been sufficiently stressful to reduce oyster feeding rates (e.g., proxy for filtration). In addition, filtration has been shown to increase with shell height (Walne, 1972), therefore the observed decrease in oyster filtration could be correlated with the decrease in oyster shell height (Figure 2).

Unlike studies such as Sanford et al. (2014) that found Olympia oysters raised under elevated CO_2_ conditions were smaller than those raised under ambient, we found no significant difference between elevated and ambient treatments for oyster shell height. This suggests that increasing temperatures can assist in offsetting the effects of acidification. Oysters also tended to have lower survival in elevated CO_2_, which is consistent with previous studies that have shown decreases in oyster survival with an increase in carbon dioxide (Talmage & Gobler, 2009). Interestingly, while we found no significant impacts of CO_2_ or temperature on several oyster traits (such as wet weight and fecal production), CO_2_ and temperature could strongly affect the strength of trophic interactions with shell crushing predators such as mud crabs.

Oysters with significantly weaker shells (Figure 5) may increase the strength of trophic interactions in oyster reef communities. In fact, a recent study demonstrated that increased acidification reduces native mud crab consumption on juvenile eastern oysters (Dodd, Grabowski, Piehler, Westfield, & Ries, 2015). However, Dodd et al. (2015) only manipulated acidification, and it is likely that the increase in the crab's metabolic rate with increased temperature could outweigh reduced foraging due to elevated CO_2_. Indeed, crab metabolism and developmental rates are known to increase with temperature (Costlow, Bookhout, & Monroe, 1962).

Overall, we found decreased growth and survival as well as less production of fecal matter (e.g., less filtration) in oysters grown in elevated temperature environments. This suggested that oysters in natural communities may see similar fitness declines with increasing temperatures. However, oysters do not live in isolation, and in the presence of important oyster predators, the combined effects of these multiple stressors could lead to significantly higher mortality rates of this important foundation species.

In concert, these data support the hypothesis that changes in temperature and CO_2_ predicted from global climate change can influence marine communities via direct effects on individual species, which could have important implications for the nature of their trophic interactions. Future research should focus on understanding the integrated effects of multiple stressors on trophic interactions. Such data will be invaluable to ecologist and managers attempting to understand and predict the impacts of climate change on important and in some cases economically valuable ecosystems.

## Conflict of Interest

The authors declare that they have no conflict of interest.

## Author Contributions

CJS and MWM formulated the question and experimental design. CJS performed the experiments. CJS and MWM analyzed the data. CJS, BRS, and MWM wrote the manuscript.
